# Predatory cues drive colony size reduction in marine diatoms

**DOI:** 10.1002/ece3.7890

**Published:** 2021-07-14

**Authors:** Kristie Rigby, Erik Selander

**Affiliations:** ^1^ Department of Marine Sciences University of Gothenburg Göteborg Sweden

**Keywords:** chemical defenses, chemical ecology, copepod, inducible defense, plankton ecology, predator–prey interactions

## Abstract

1. Colony formation is a common feature among nonmotile marine phytoplankton. Several theories exist around the potential benefits of larger colonies.
2. Here, we test the hypothesis that predation is one of the drivers behind colony formation and chain length plasticity. We exposed cultures of *Thalassiosira rotula*, *Chaetoceros curvisetus*, and *Chaetoceros affinis* to copepodamides, a chemical alarm cue released by copepods and perceived as an indicator of predation threat by their prey. This was coupled with a grazing experiment, which compared copepod grazing rates on different chain lengths.
3. Our results show that *T. rotula* and *C. curvisetus* decreased their chain lengths by 79% and 49%, respectively, in response to copepodamides. Single cells and short chains were grazed at lower rates compared with long chains, and the copepodamide‐driven size shift led to 30% and 12% lower grazing in *T. rotula* and *C. curvisetus*, respectively. In contrast, *C. affinis* showed a slight increased chain length in response to copepodamides although nonsignificant.
4. We found that 2 of 3 studied species reduce their chain length in response to the presence of copepod grazers. Altered size structure has implications for the route of carbon in the marine food webs and carbon export to deeper strata.

## Graphical abstract

Here, we test the hypothesis that predation is one of the evolutionary drivers behind chain formation and chain length plasticity. We expose three common species of chain‐forming marine phytoplankton to isolated alarm cues from zooplankton grazers, copepodamides. Two out of three chain formers responded in the predicted way and shorten their chains (by 79% and 49%, respectively). Moreover, we show in grazing experiments that the smallest colonies formed are almost completely protected from grazing.

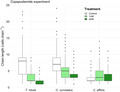

## 1 INTRODUCTION

Predation is the largest mortality factor for marine phytoplankton. In order to minimize the risk of predation, a wide range of anti‐predatory strategies have evolved. Some examples of these adaptions involve nocturnality, camouflage, toxin production, mimicry, and various behavioural strategies. Colony formation is one antipredator behaviour in which organisms will form colonies to increase, or decrease, in size beyond the handling capacity of the predator (Lürling, 1999; Lürling & van Donk, 1997).

Colony size is under selection by more factors than predation. The microscopic cyanobacteria *Trichodesmium* shuffles iron into the colony core, thereby protecting it from loss (Rubin et al., 2011). Yet, there are some fundamental trade‐offs associated with colony formation; larger colony size comes with higher encounter rates with enemies (Selander et al., 2011). Furthermore, colonies have to share resources and also run higher risk of sharing diseases (Kenitz et al., 2020). In the microscopic marine environment, colony formation in phytoplankton is very common, especially in cyanobacteria, green algae, dinoflagellates, and diatoms (Hessen & Van Donk, 1993; Rippka et al., 1979; Smayda, 2010). Diatoms are one of the largest groups of phytoplankton, often forming long chains which plays a significant role in the cycling of oxygen and carbon from the earth's atmosphere (Behrenfeld, 2021).

**FIGURE 1:**
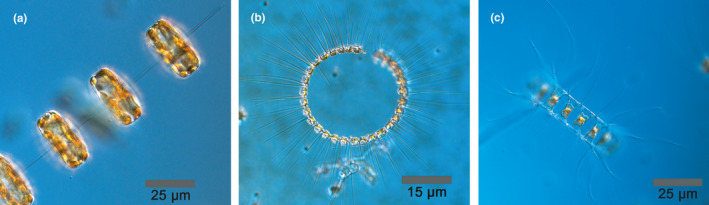
Chain‐forming diatoms (a) *Thalassiosira rotula*, (b) *Chaetoceros curvisetus*, and (c) *Chaetoceros affinis*. Photos: Ann‐Turi Skjevik, SMHI

Diatoms have relatively fast growth rates compared with other phytoplankton taxa and can dominate over other protists in low light settings and temperatures (Raven & Geider, 1988). The total biomass of diatoms totals to around 0.1% of that of terrestrial plants (Bar‐On et al., 2018; Leblanc et al., 2012). Yet, diatoms contribute one fifth of the global primary production (Nelson et al., 1995). Diatom carbon fixation consequently equals the total carbon emissions from fossil fuels, ~10 Gt C y^−1^ (Le Quéré et al., 2015; Longhurst et al., 1995). In the marine environment, it has been estimated that diatoms supply up to 45% of the total primary production (Mann, 1999), and they flourish in aquatic environments by making the most from their vacuoles, buoyancy regulation, and luxury nutrient uptake (Behrenfeld, 2021; Hansen & Visser, 2019). Chain formation is one of the more conspicuous traits they possess and is speculated to be driven by predation, although this is still debated (Behrenfeld, 2021; Verity & Villareal, 1986; Figure 1). Chains are typically formed with silica or chitin strands between daughter cells, accompanied by mitotic division (Round et al., 1990; Young et al., 2012). Chain length is, however, a plastic trait. Long chains can be shortened by physical stress from, for example, intense turbulence (Lovecchio et al., 2019) or as a response to nutrient limitation (Pahlow et al., 1997; Takabayashi et al., 2006).

Several theories exist around the costs and benefits of chain formation. It has been suggested that chain formation is favoured under nutrient‐replete conditions, as cells within chains do not have to compete for nutrients (Takabayashi et al., 2006). Along with predation risk, nutrient availability, self‐shading, and light come at a fitness cost for chain formers as they need to share their resources with their neighbouring cells (Karp‐Boss & Boss, 2016; Karp‐Boss et al., 1996). As nutrients become limiting, however, cells in chains will deplete the near field nutrient concentrations and thereby reduce the flux of nutrients to neighbouring cells. Turbulence may reduce the intra‐chain competition for nutrients by increasing nutrient flux to cells in chains but single cell phenotypes still outperform chain‐forming phenotypes when it comes to nutrient uptake (Pahlow et al., 1997). This was later confirmed experimentally by Bergkvist et al. (2018). Pahlow et al. (1997) conclude that the evolution of chain formation was likely driven by other factors than competition for nutrients.

On a global scale, it is estimated that 12% of the pelagic primary production is consumed by mesozooplankton (Calbet, 2001) and 67% by microzooplankton (Calbet & Landry, 2004). As consumption is both intense and size selective, predation is likely a strong evolutionary driver selecting for differences in size. By adapting colony size, microalgae can escape predation, thereby reducing overall population losses (Hay, 2009; Hay & Kubanek, 2002; Jakobsen & Tang, 2002; Long et al., 2007). In addition, splitting colonies into smaller units reduces the cell‐specific encounter rate for both motile and nonmotile chain formers from predation (Bergkvist, 2012; Bjærke et al., 2015; Selander et al., 2011). Finally, increased chain length has also been suggested to be an adaptation to reduce sinking rates (Smayda & Boleyn, 1966) allowing diatoms to stay in the euphotic zone. However, this only holds true if the diatoms are alive, dead colonies tend to sink faster than single cells (Waite et al., 1997).

No single factor has been shown to be the driving force of chain length evolution. However, recent studies have shown that colony size is reduced in response to grazer cues in *Skeletonema marinoi* and the colony‐forming *Phaeocystis* spp which adjusts colony size to minimize loss to prevailing predators (Amato et al.,^2018^; Bergkvist et al., 2012; Long et al., 2007). Long et al. (2007) report a decrease in colony size in response to large grazers, but an unaltered or increased colony size in response to smaller microzooplankton. In both cases, the change is accompanied by reduced grazing losses. Furthermore, micro‐ and mesozooplankton are often negatively correlated in the northeast Atlantic, as mesozooplankton suppress microzooplankton which makes switching between small and large colony size beneficial (Bjærke et al., 2015; Hansen et al., 1994).

Copepods induce a variety of defensive traits in phytoplankton. Some examples include slower swimming speeds, increased toxin content, increased bioluminescence capacity, and reduced chain length (Lindström et al., 2017; Selander et al., 2011, 2015). A group of polar lipids which are exuded by copepods, copepodamides, have been identified as grazer cues for phytoplankton organisms. Copepods taint the water with this chemical marker which cues the phytoplankton to initiate their defensive traits in efforts to avoid being eaten (Selander et al., 2019). Although copepodamides have been shown to induce chain length shortening in *Skeletonema marinoi* (Selander et al., 2019), it is unknown if other taxa/species of chain‐forming diatoms respond with the same defensive mechanism. Chain formation plays a pivotal role related to processes involving the global carbon cycle such as trophic transitions, aggregate formation, vertical transport of organic matter, and silica (Bergkvist et al., 2018). Diatoms fixate inorganic carbon in the photic zone and transfer this to the seafloor mainly through sedimentation of fast sinking aggregates, “marine snow,” or faecal pellets from grazing organisms. If other chain‐forming diatoms also reduce size in response to copepod cues, the altered size distribution may affect the route of elements in biogeochemical cycles.

Here, we test the hypothesis that predation is an important driver behind chain formation and chain length plasticity. We exposed 3 globally occurring marine diatoms, *Thalassiosira rotula*, *Chaetoceros curvisetus*, and *Chaetoceros affinis* to copepodamides, and perform controlled grazing experiments with the induced phenotypes to evaluate possible fitness benefits associated with chain length plasticity.

## 2 MATERIALS AND METHODS

### 2.1 Copepodamide experiment

Stock cultures of *T. rotula* strain CCAP1085/20, *C. curvisetus* strain RCC6895, and *C. affinis* strain CCAP1010/27 were grown at 16°C in f/2 enriched with silica media (Guillard & Ryther, 1962) at 26 PSU salinity. All algae strains were obtained from Gothenburg University Marine Culture Collection, Sweden. A well‐mixed culture was diluted to 100 cells mL^−1^ in f/4 media with silica (Guillard & Ryther, 1962) and divided into 8.5 mL glass vials with 5 replicates per treatment. The vials were pre‐treated with copepodamides corresponding to a concentration of 0, 1, or 5 nM. Copepodamides were added dissolved in methanol and the solvent evaporated under a stream of N_2_ before the culture was added. Controls received the same volume of methanol without copepodamides. Copepodamides were extracted from freeze‐dried *Calanus finmarchicus* as per: Selander et al. (2015). The vials were filled and placed on a plankton wheel with a rotation of 0.5 rpm. Copepodamides slowly dissolve from the vial wall and degrade over time. The average effective concentration is 1%–2% of the nominal concentration corresponding to ~10 pM and 55 pM (Selander et al., 2019). The vials were incubated for 48 hr at 16°C with a 16:8, light:dark cycle approximately 120 f molm^−2^ s^−1^. A 1 mL well‐mixed sample was gently pipetted to a 48‐well plate where the length of the first observed 100 chains in a random location was recorded in each replicate. Cell concentrations were determined with a 1 mL Sedgewick Rafter Counting Chamber where at least 0.1 mL per replicate was counted. Growth rates (µ, d^−1^) were calculated as

(1) $$\mu =\frac{\text{LN}\left(C_{2}\right)‐\text{LN}\left(C_{1}\right)}{\Delta t}$$

where *C*_1_ and *C*_2_ are the concentrations at the start and end of the experiment, Δ*t* is the elapsed time between the two time points.

### 2.2 Grazing experiment

We grew stock cultures with and without copepodamide additions administered as above (1 nM every 48 hr over a 7‐day period). In order to assess grazing rates on both grazer induced (shorter chains) and control (longer chains), we mixed both cultures in equal proportions and diluted to a final concentration of ~200 µg C L^−1^ (Strathmann, 1967) in f/10 media enriched with silica (Guillard & Ryther, 1962). The mixture was divided between 8 bottles (310 mL glass bottles) with 4 bottles receiving 8–10 adult or late copepodite stage *Acartia tonsa* (prosome length ~700 µm). Bottles were then incubated on the plankton wheel for 20 hr with the same conditions as the copepodamide experiment. Individual chains were counted and sized (biovolume µm^3^) at the start and end of the experiment with a Beckmann Coulter Counter Multisizer 3. Clearance rate (*F*) was calculated for each size interval according to Frost (1972):

(2) $$F=\frac{V}{\mathrm{tn}}\ln\left(\frac{C_{g}}{C_{c}}\right)$$

where *V* is the volume of the bottle (mL); *t*, the incubation time (h); *n*, the number of copepods; and *C*_c_ and *C*_g_, the biovolume per mL (µm^3^ mL^−1^) in grazer‐free (control) and grazed bottles, respectively. For comparison of total losses to grazers on a diatom population level, the size‐specific clearance rate was multiplied with the proportion of the population being of that size interval and integrated for the size distribution of control, or copepodamide‐induced cultures obtained by coulter counts.

### 2.3 Statistical analysis

Chain length from the copepodamide experiment was compared using a generalized linear mixed model (GLMM) with a Poisson distribution as the chain lengths were recorded as count data and not normally distributed. In the model, copepodamide concentrations are a fixed effect with 3 levels. 100 individual chain lengths were measured from each replicate, and since these were not independent, replicate was nested as a random effect under treatment (Table. S1). Growth rates (µ) were compared using a one‐way analysis of variance (ANOVA) with a Tukey's post hoc test to test whether growth was significantly affected by the addition of copepodamides. The size dependence of clearance rate was tested by a linear regression with clearance rate as dependent variable and size (equivalent spherical diameter) from the coulter counts as the independent variable. Equivalent spherical diameter (ESD) scales to the third root of the volume, and hence, larger ESD is equivalent to longer chains.

All analyses were performed using R software, version 3.6.2 and packages lme4, lmerTest, and nlme (Bates et al., 2015; Pinheiro J et al., 2020).

## 3 RESULTS

**FIGURE 2:**
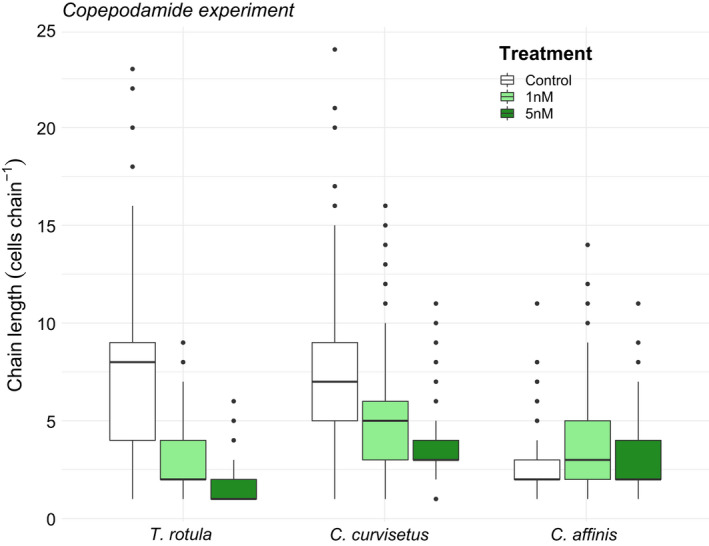
Boxplots showing chain length of three species of chain‐forming diatoms (*T. rotula*, *C. curvisetus*, and *C. affinis*) after 48 hr of exposure to one of three treatments (control, 1 nM, or 5 nM of added copepodamides). In total, 500 observations of chain lengths were made per species and treatment (5 replicates of 100 observations). Solid line inside the box signifies the median, box contains the lower and upper quartile ranges, and dots represent outliers

**FIGURE 3:**
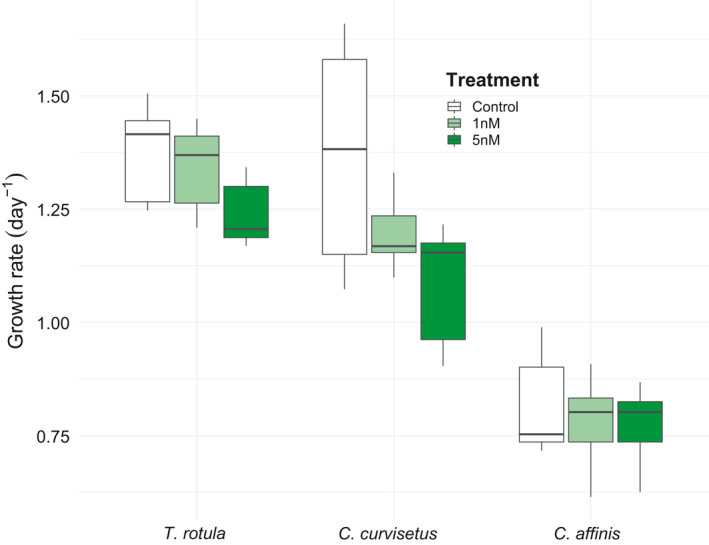
Box plot showing the specific growth rates (day^−1^) for *T.rotula*, *C. curvisetus*, and *C. affinis* from the copepodamide experiment. Each species has 5 replicates per treatment. Solid line inside the box signifies the median, and box signifies the lower and upper quartile ranges

*T. rotula* and *C. curvisetus* exposed to copepodamides significantly reduced their chain lengths compared with the controls (Table. S1, Figure 2). *T. rotula* chain length decreased by 79% in the 5 nM treatment compared with the control and 64% in the 1 nM when compared to the control (*p* < 0.001). *C. curvisetus* chain length reduced by 49% in the 5nM and 28% in the 1nM (*p* < 0.001). There was no significant effect of copepodamide treatment for *C. affinis* (*p* = .85) although the average chain length increased by 15% in 5nM and 46% in 1nM (Table. S1, Figure 2). Even though there was a trend toward a reduced total cell count in *T. rotula* and *C. curvisetus*, the reduction was not significant, indicating little or no inhibition of growth from copepodamides (Figure 3, *T. rotula p* = 0.12, *C. curvisetus p* = 0.07, *C. affinis p* = 0.76).

**FIGURE 4:**
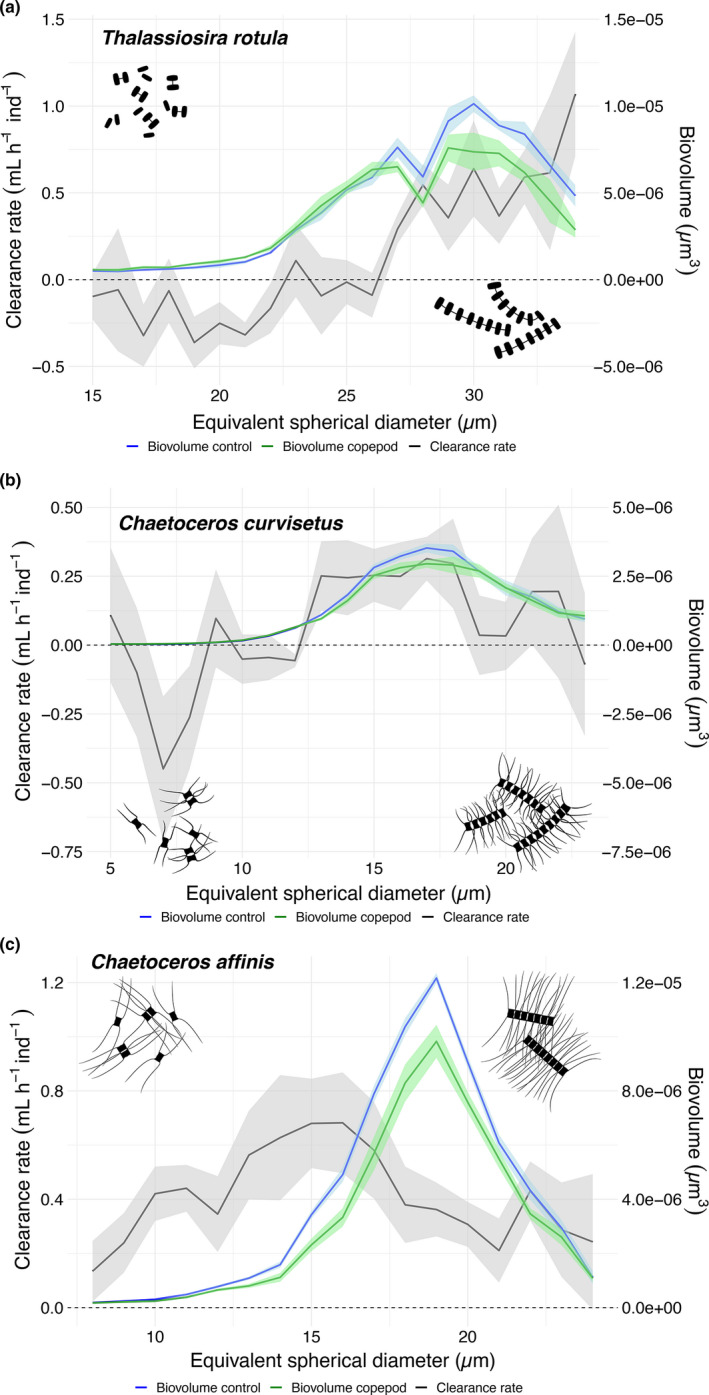
Copepod clearance rates and size distribution of (a) *T. rotula*, (b) *C. curvisetus*, and (c) *C. affinis*, plotted against prey sizes (equivalent spherical diameter). Solid black line displays the mean clearance rate, calculated as volume swept clear per hour per individual copepod (mL h^−1^ ind^−1^) for each size class, from four replicates with standard error shaded in grey. Black dashed line represents neutral effect of copepods, that is, no grazing. Green and blue lines display the size distribution (biovolume) of particles from grazed cultures and non‐grazed cultures (control) at the end of the experiment

In the grazing experiments, where copepods were offered a broad distribution of different chain lengths, grazing rate was positively correlated to chain length in *T. rotula* (*R* ^2^ = 0.73; *p* ≤ 0.001) and *C. curvisetus* (*R* ^2^ = 0.18; *p* = 0.04), but not *C. affinis* (*R* ^2^ = −0.06; *p* = 0.73, Figure 4, Figure S2). This result coincides with the copepodamide experiment results where a reduction in chain length was not observed in *C. affinis* (Figure 2). For *C. curvisetus*, clearance decreased again for the largest colonies (Figure 4b). Total grazing rate, integrated across all size classes, was 30% and 12% lower in copepodamide‐induced cultures for *T. rotula* and *C. curvisetus*, respectively.

## 4 DISCUSSION

Our results show that two of the three species tested, respond to copepodamides by decreasing their chain lengths (Table. S1, Figure 2). In the grazing experiment, the clearance rate for *T. rotula* and *C. curvisetus* was positive for larger size classes and negative for smaller size classes (Figure 4a,b), thereby confirming that grazing pressure is lower on the grazer‐induced phenotype with shorter chains (Bergkvist et al., 2012). Overall, *T. rotula* had a larger reduction in chain shortening (79%) compared with the in *C. curvisetus* (49%). These percentages are in line with the previously observed shortening in *Skeletonema marinoi* from Selander et al. (2019) which showed a chain length reduction of 50%.

*C. curvisetus* is a smaller species with an apical axis of 7.5–12.5 µm (Lee et al., 2014) compared with *T. rotula* at 17.5–25 µm (Tomas et al., 1996). While *A. tonsa* can feed on particles as small as 5–10 µm ESD, they feed more efficiently on larger particles (Berggreen et al., 1988). Thus, the decreased clearance rate on single cells and shorter chains is in agreement with other studies on size‐dependent clearance of particles in copepods.

*C. affinis* did not respond with a reduction in chain length from exposure to copepodamides. Thus, this species might rely on other traits to endure copepod grazing. The morphology of the setae (bristle‐ or hair‐like structure) differs between these *Chaetoceros* species. The terminal setae are very thick on *C. affinis*, in contrast to the much thinner setae on *C. curvisetus* (Lee et al., 2014). The setae thickness may pose as another defence mechanism, by being more rigid and perhaps harder to capture and digest. High magnification slow motion video up‐takes would be a way to explore this hypothesis. It is interesting to note that grazing was not as clearly size dependent in *C. affinis* which was the only species that did not respond to copepodamides by chain length shortening (Figures 2 and 4c).

The grazing experiments may be partly confounded by copepods actively fragmenting chains. This would lead to the same pattern, with lower, or even negative clearance in shorter units and higher in the larger size classes. Similarly, the copepods will exude copepodamides that could also potentially contribute to the break‐up of longer chains, and falsely lead us to conclude that grazing rates are higher for the larger size classes. However, the grazing experiments were done overnight, which is shorter than the time required for grazer‐induced chain length shortening to develop (Bergkvist et al., 2012). In addition, the higher clearance rate on larger food items is consistent with copepod feeding biology, driven both by poor retention of smaller particles and by the inherently higher encounter rates of larger food items (Berggreen et al., 1988; Selander et al., 2011). The experiments by Berggreen et al. (1988) were performed with single‐celled prey of different sizes, and hence cannot be confounded by fragmentation. It is still possible that physical fragmentation and/or grazer induced chain length shortening contributed to the size‐selective grazing result, but the size distribution (Figure 4a‐c) before and after grazing together with the short duration of the grazing experiment suggests that this is likely a minor part of the observed difference in clearance rate and that removal of larger particles is the main reason.

The current experiment was carried out in a simplified environment with single species laboratory cultures. In nature, phytoplankton face a more complex and diverse grazing community with both smaller (microzooplankton) feeding and larger (mesozooplankton) feeding on the phytoplankton simultaneously. Since the microzooplankton typically feed on smaller food items than mesozooplankton (Hansen et al., 1994), breaking up chains could be counterproductive by exposing the smaller units to the more plentiful microzooplankton grazers (Hansen et al., 1994). Yet the microzooplankton are typically negatively correlated with mesozooplankton, such as copepods, as mesozooplankton have high clearance rates on microzooplankton (Bjærke et al., 2015). Thus, chain‐forming diatoms similar to other colony‐forming phytoplankton, for example, *Phaeocystis* spp. probably benefit from adjusting their colony size to minimize losses to the current grazer regime. In the case of *Phaeocystis*, it has been shown that larger colonies are formed in response to ciliate grazers and smaller in response to copepod grazers (Long et al., 2007). Using natural concentrations of meso‐ and microzooplankton grazers, Bjærke et al. (2015) calculated that grazer induced colony size plasticity, reduce losses to grazers by 31% and 36% compared with fixed single cell, or fixed long‐chain strategies, respectively.

Similarly, the influence copepodamides have on diatom morphology and colony size may have profound effects on large‐scale processes, such as pelagic food size structure and on the route of carbon in the pelagic food web. Shorter colonies are likely to slow down the carbon export as they take longer until they encounter other chains, form aggregates, and sink out, transporting carbon to the ocean interior. Long chains will aggregate faster with other particles as the encounter rate is much higher (Alldredge & Gotschalk, 1988; Jackson, 1990). Short chains are often grazed by microzooplankton, which in turn are consumed by copepods and other mesozooplankton. In contrast, longer chains are often directly grazed by copepods and other mesozooplankton. Thus, chain length plasticity in diatoms has the capability to determine and alter the length of the food web (Stibor et al., 2004), and subsequently the biogeochemical cycling of elements and export to deeper strata. Understanding these environmental cues can expand our knowledge of the functioning of the marine ecosystem and provide further insights into how indirect predatory cues can drive changes in the food web.

## CONFLICT OF INTEREST

None declared.

## AUTHOR CONTRIBUTIONS

**Kristie Rigby:** Conceptualization (lead); Data curation (lead); Formal analysis (lead); Visualization (lead); Writing – original draft (lead); Writing – review and editing (equal). **Erik Selander:** Conceptualization (supporting); Data curation (supporting); Formal analysis (supporting); Visualization (supporting); Writing – original draft (supporting); Writing – review and editing (equal).

## Supporting information

Supplementary Material

## Data Availability

Data for this study are available from the Dryad Digital Repository as follows: Rigby_&_Selander_2021_Data submitted with DOI https://doi.org/10.5061/dryad.4xgxd2598.
