# Supplementary material for: Predatory cues drive colony size reduction in marine diatoms
Source: Ecol Evol. 2021 Jul 14;11(16):11020–7. doi: 10.1002/ece3.7890 (PMC8366847; doi:10.1002/ece3.7890)
Supplement: Supplementary file 1 — Supplementary Material [file ECE3-11-11020-s001.pdf]

Supplementary

Table. S1. Results of a generalized linear mixed model (GLMM) of changes in chain length with exposure to copepodamide treatment. The model indicates *T. rotula* and *C. curvisetus* were significantly shorter in the copepodamide treatment. The model has Poisson distribution with treatment as a fixed factor and replicate as a random factor nested within treatment. SE= standard error, Z value = parameter estimated. Total number of observations per treatment 1500. Significance codes: 0 '***' 0.001 '**' 0.01 '*' 0.05 '.' 0.1 ' ' 1.

**Species**		**Estimate**	**S.E**	**z value**	**Pr(>|z|)**	**Variance**	**S.D**
T. rotula							
Fixed effect	(Intercept)	1.717	0.119	14.317	< 2e-16 ***		
	Treatment	-0.266	0.041	-6.481	9.11e-11 ***		
Random effect	Replicate: Treatment					0.114	0.337
C. curvisetus							
Fixed effect	(Intercept)	1.846	0.036	51.735	< 2e-16 ***		
	Treatment	-0.123	0.012	-9.823	< 2e-16 ***		
Random effect	Replicate: Treatment					0.008	0.092
C. affinis							
Fixed effect	(Intercept)	1.108	0.058	18.976	< 2e-16 ***		
	Treatment	0.003	0.019	0.196	0.845		
Random effect	Replicate: Treatment					0.024	0.155

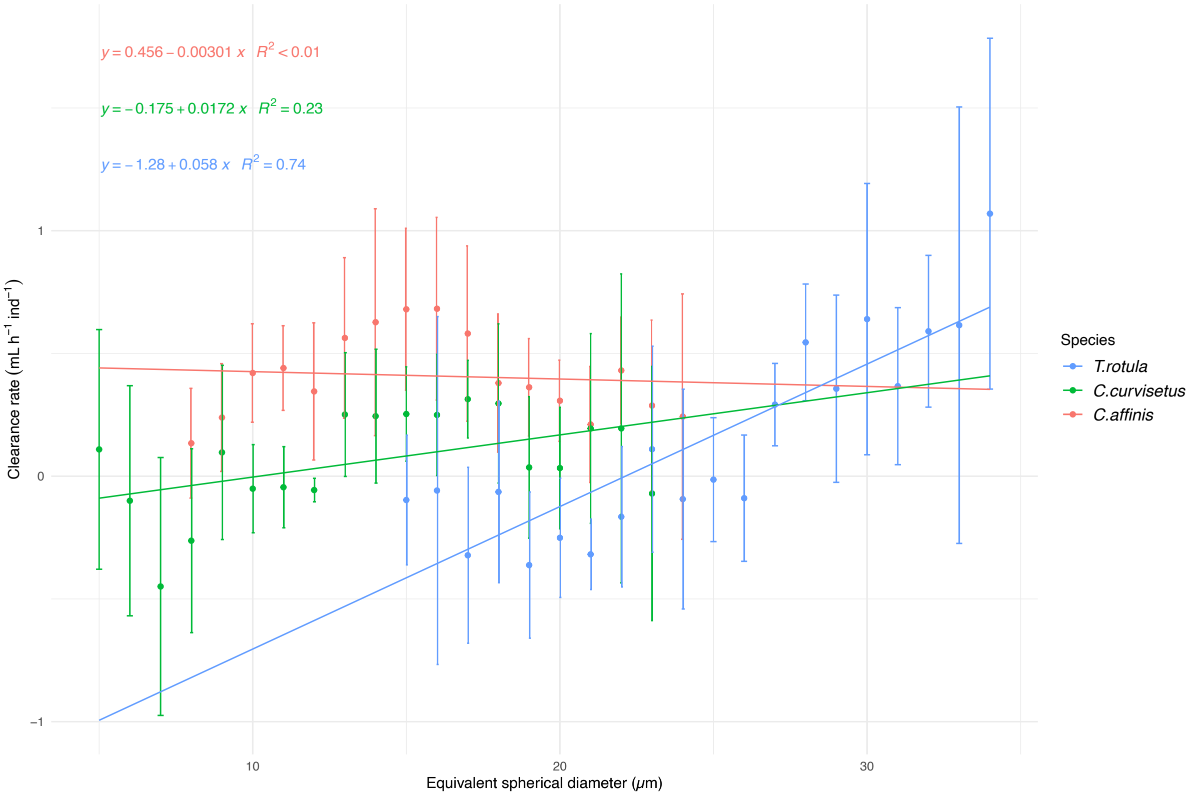

Figure S2: Simple linear regression for *T. rotula*, *C. curvisetus* and *C. affinis* from the grazing experiment. Mean clearance rate is plotted as points for each ESD value with +/- SD error bars with a linear trendline fitted *n*=4.
